# Biogas Digestate and Its Electrodialysis Concentrate as Alternative Media Composition for *A. platensis* Cultivation: A Study on Nutrient Recovery from Dairy Wastewater

**DOI:** 10.3390/bioengineering12050460

**Published:** 2025-04-26

**Authors:** Elena Singer, Sun-Hwa Jung, Vivekanand Vivekanand, Christoph Lindenberger

**Affiliations:** 1 Department of Mechanical Engineering/Environmental Technology, Ostbayerische Technische Hochschule Amberg-Weiden, 92224 Amberg, Germany; s.jung@oth-aw.de (S.-H.J.); c.lindenberger@oth-aw.de (C.L.); 2 Centre for Energy and Environment, Malaviya National Institute of Technology Jaipur, Jaipur 302017, India

**Keywords:** wastewater treatment, cyanobacteria, microalgal cultivation, wastewater-based media

## Abstract

The dairy industry generates substantial nutrient-rich wastewater, posing environmental challenges if discharged untreated. This study explores the potential of using the cyanobacterium *Arthrospira platensis* for nutrient recovery from dairy wastewater, precisely the liquid biogas digestate (BD). The research investigates the feasibility of utilising BD and electrodialysis-concentrated BD (BD concentrate) as alternative media for *A. platensis* cultivation, with a focus on biomass productivity, nutrient uptake, and high-value product formation. Batch and continuous cultivation modes were employed. In batch experiments, biomass productivity was in the ratio of 0 and 0.27 g L^−1^ d^−1^, which was 8–100% lower than simulated values for all five tested media compositions. Phosphate fixation was limited with no fixation during batch cultivation and 8–69% during continuous cultivation, likely due to suboptimal N/P ratios, while ammonium removal remained consistently high (>98%). Phycocyanin yield decreased significantly by 92% at high BD concentrate concentrations compared to standard media. Continuous cultivation with 50% BD concentrate improved biomass productivity to 1.02 g L^−1^ d^−1^ and pigment yield to 107.9 mg g^−1^, suggesting a sufficient supply of nutrients. The findings highlight the potential of BD-based media for nutrient recovery but emphasise the need for optimisation strategies, such as nutrient supplementation and microbial adaptation, to enhance performance.

## 1. Introduction

The dairy industry produces a substantial volume of nutrient-rich wastewater. In the European Union alone, the dairy industry generates approximately 192.5 million cubic metres annually and is the largest wastewater producer in the food industry [1,2]. The treatment of this wastewater is an energy-intensive process, resulting in a significant financial burden. The cost per cubic metre of treated wastewater is approximately 1.21€ [2]. Meanwhile, the world’s population is increasing exponentially and is expected to reach 9.71 billion by the year 2050. Subsequently, the wastewater generation from private and industrial sectors will increase with the increase in demands of fresh water, while the fresh water resources are limited [1,3]. Additionally, the direct discharge of wastewater without any treatment causes water pollution, environmental and health problems [1]. For instance, soil salinisation is a pressing ecological concern that stands as one of the most significant environmental risks of the 21st century. This degradation of soil quality, largely exacerbated by modern agricultural practices and climate change, threatens ecosystems, agricultural productivity, and long-term sustainability [4,5,6]. The process is driven by rapid salt dissolution in soil, which migrates through soil capillaries to the surface, where it evaporates and concentrates salts [7].

Moreover, the discharge of phosphorus (P) and nitrogen (N) rich wastewater into water bodies or its utilisation as fertilisers causes eutrophication, acidification, and the release of greenhouse gases like nitrous oxide—a highly potent climate-warming agent [7,8]. Consequently, anthropogenic eutrophication causes an imbalance within water bodies in nutrients. Due to the high availability of nutrients, the phenomenon of algal blooms has been observed, causing high water turbidity, low light conditions, and high concentrations of dissolved nutrients [9].

Subsequently, the dairy industry faces significant environmental challenges that strongly necessitate improvements in wastewater management and its treatment in order to mitigate potential hazards. According to the conventional wastewater treatment system, it undergoes a three-step treatment process: primary treatment removes settleable impurities, secondary treatment removes biodegradable organic matter, and tertiary treatment purifies the water to meet specific requirements [10,11]. The third step has its own significance in preventing eutrophication or soil salinisation by the removal of significant quantities of N and P sources [12,13,14]. However, conventional methods like chemical processes are costly as they have a high demand for energy and chemicals. Furthermore, only a minority of these methods can recover nutrients like ammonium and phosphate [14,15,16]. In addition, the conventional methods like chemical processes for removing nitrogen, conversion of N to gas and phosphate-enriched sludge are expensive to operate, have a high energy demand, need regular maintenance in short time intervals, and they sometimes fail to meet the discharge standards [10,14,16,17]. This underscores the importance of nutrient recovery as the phosphorus resources are diminishing, and its scarcity necessitates more efficient recycling approaches [12,13]. Consequently, alternative methods like microalgae-based systems for nutrient recovery are being investigated currently [18,19,20].

The cultivation of microorganisms like cyanobacteria and microalgae on nutrient-rich wastewater, such as dairy wastewater or liquid fermentation residue, has emerged as a promising strategy for nutrient recovery [19]. Unlike traditional wastewater treatment processes, which require high energy inputs, cyanobacteria utilise sunlight as an energy source to drive photosynthesis, enabling biological conversion of nutrients into biomass [18,21]. Different approaches have been explored for microalgae-based wastewater treatment, including monocultures of specific strains like *Scenedesmus* and *Chlorella vulgaris* [19,22], as well as consortia of multiple microalgae species [23] or even microalgae-fungus systems [18,24], which can enhance nutrient removal efficiency and biomass productivity. Therefore, the present study explores the use of the cyanobacterium *Arthrospira platensis*, chosen for its versatile metabolic system, which enables it to adapt to various environmental conditions and exploit available nutrients for growth [25].

Thereby, wastewater treatment and microalgae-based nutrient recovery bring various advantages. Firstly, microorganisms can remove different pollutants like phosphate and ammonium at the same time. Additionally, it is possible to lower the concentrations to a low level which is beneficial to meet required standards. The produced biomass can be used for different purposes like a carbon-neutral fuel, fertilizer or high value products like pigments and sugars can be extracted. The environmental impact of the cultivation of microalgae can potentially be lowered as fewer chemicals and freshwater are needed for the media [10,17,26]. Cyanobacteria cultivation not only reduces the salt content in wastewater but also produces valuable biomaterials, aligning with the principles of a circular economy.

This study investigates the use of liquid biogas digestate (BD) from dairy wastewater as a nutrient source for *A. platensis* cultivation, incorporating electrodialysis as a pretreatment step to selectively isolate key nutrients and mitigate salinity-related growth limitations. The study assesses the performance of *A. platensis* in both batch and continuous cultivation using different BD-based media, including electrodialysis-treated variants. The study evaluates nutrient recovery efficiency alongside the production of the two high-value bioproducts phycocyanin and exopolysaccharides which have promising market potential and applications. A preliminary cost assessment further compares the economic feasibility of chemical additions to BD media against product yields. The novelty of this work lies in leveraging electrodialysis for BD valorisation, offering a sustainable alternative for wastewater treatment while enhancing biomass productivity and product yield.

## 2. Materials and Methods

### 2.1. Characterisation and Pretreatment of Biogas Digestate

The liquid fermentation residue was collected from a biogas plant run by the private dairy Bechtel in Schwarzenfeld, Germany. The composition of the biogas digestate and the electrodialysis concentrate in terms of phosphate, ammonium, and nitrate is summarized in Table 1. Prior to the execution of the experiments, the BD was pretreated with a milk centrifuge Milky FJ 125 EAR3 from Janschitz GmbH, Althofen, Austria and filtrated (4–12 µm) to remove solid particles. The liquid fermentation residue was autoclaved to prevent bacterial contamination and used directly or processed via electrodialysis to generate a concentrate (BD concentrate) with the phosphate and ammonium concentrations presented in Table 1. However, the phosphate and ammonium from the biogas digestate were not completely transferred to the BD concentrate, resulting in a transfer efficiency of less than 23%.

#### Electrodialysis Treatment of Biogas Digestate

The electrodialysis process was applied to selectively transfer nutrients such as ammonium and phosphate from the biogas digestate into the concentrate stream using ion-exchange membranes. This resulted in a nutrient-rich, low-turbidity solution suitable for microalgal growth, reducing potential inhibition and improving light penetration during cultivation. Hereby, the Electrodialysis laboratory plant BEA05, Hescon GmbH, Engstingen, Germany with fumasep^®^ FKB-PK-130 cation exchange membrane and fumasep^®^ FAA-3-PK-130 anion exchange membrane from FuMA-Tech, Bietigheim-Bissingen, Germany was utilised. During the process, the conductivity and pH value were monitored. The process was stopped when the biogas concentrate reached a conductivity of approximately 8200 µS cm^−1^. The biogas digestate concentrate was again autoclaved and then used for the cultivation of the microorganism *A. platensis*.

### 2.2. Cyanobacterial Strain and Experimental Setup

The cyanobacterium *Arthrospira platensis* (NES-39) was cultivated in Zarrouk’s nutrient-rich media (SOT) according to the formulation recommended by NIES [27]. The preculture was cultivated in a shake flask with a liquid height of approximately 5 cm (T = 22 ± 2 °C, PFD = 50 μmol m^−2^ s^−1^). The full composition of SOT medium is provided in Table 2.

### 2.3. Flask Batch Experiment and Continuous Experiment

Flask experiments were conducted in 500 mL and 250 mL flasks in triplicates (*n* = 3). The media was inoculated with the subculture and the OD of 0.1 (biomass concentration of 0.05 g L^−1^) was set. The cultures were placed on a shaker (75 rpm) (PROMAX 1020, Heidolph Scientific Products GmbH, Schwabach, Germany), at 34 ± 2 °C, and a light intensity of 68.1 μmol m^−2^s^−1^. The OD and pH measurements were taken daily for 14 days using a Spectrophotometer UV 7 (Mettler Toledo, Columbus, OH, USA) and a WTW Multi 9620 IDS + SenTix 940 (Xylem Analytics Germany Sales GmbH & Co. KG, Weilheim, Germany), respectively. The phosphate and ammonium content of the media was measured before and after the experiment period. Additionally, the biomass productivity, phycocyanin (PC), and exopolysaccharides (EPS) were measured after the experiment. Three different media compositions were tested and compared with SOT media: (1) Mixing of 25% BD and 75% SOT, (2) Mixing of 12.5% BD and 87.5% SOT, (3) Mixing of 5% BD and 95% SOT.

**Figure 1:**
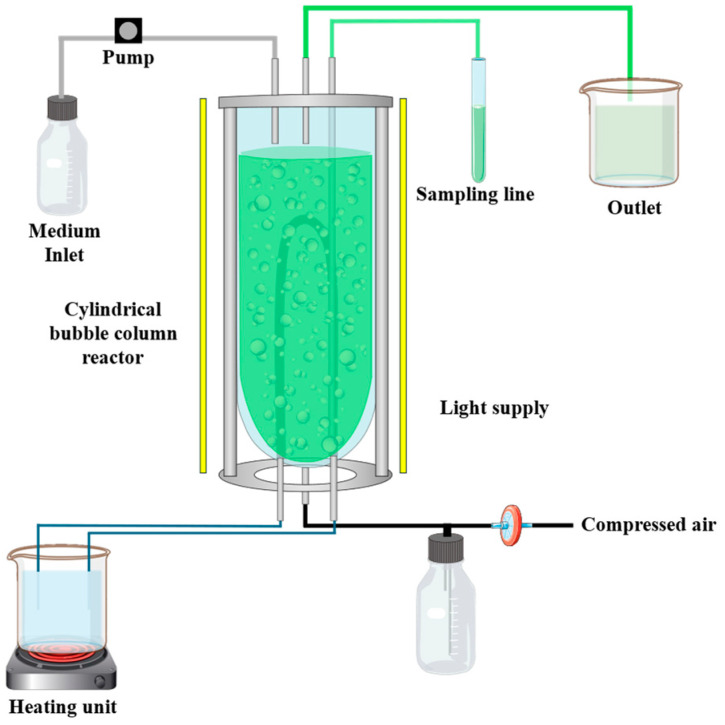
Experimental setup of cylindrical bubble column reactor system for the continuous cultivation.

Furthermore, two media compositions based on BD concentrate were evaluated in a continuous process in duplicates (*n* = 2): (1) Mixing of 50% BD concentrate and 50% SOT media, and (2) 100% BD concentrate with 16.8 g L^−1^ NaHCO_3_. The latter necessitates an additional carbon source because during the anaerobic digestion process, methane is produced, consequently resulting in a lack of carbon sources in the liquid fermentation residue. Therefore, cylindrical bubble column reactor systems (Figure 1) with a volume of approximately 820 mL (diameter = 5 cm) were inoculated with the subculture and an OD of 0.1 (biomass concentration of 0.05 g L^−1^). During the experimental time of 30 days, the aeration with compressed air and temperature were kept constant at approximately 60 L h^−1^ and 35 ± 2 °C, respectively. The light was supplied continuously for 24 h from three directions and set at a value of approximately 180 μmol m^−2^ s^−1^. Hereby, the intensity of the light was measured at nine different points on the surface of the reactor using the light intensity sensor LI-250A from LI-COR, Lincoln in Nebraska, USA. In addition, the pH value and OD were measured by taking a sample of 4 mL daily. The volume of the reactor systems was controlled by an adjustable feed pump and a level controller. In this study, it was investigated if the cyanobacterium *A. platensis* can be cultivated in a continuous process on the two media compositions based on the BD concentrate, and the EPS and phycocyanin yield of this setup was compared with data from the literature.

### 2.4. Analytical Methods

The biomass concentration was determined to monitor the growth rate of the cultures. Therefore, the OD value was measured at 750 nm. The growth medium was used as a blank. The wavelength was chosen to avoid interference from other light-absorbing cellular pigments like chlorophyll. The OD values were calibrated to the dry biomass weight.

To complement the experimental results, biomass growth was simulated using the light-dependent growth model proposed by Jung et al. [28]. This model describes the relationship between biomass concentration (X), the growth-inhibiting constant for surrounding biomass (*K*_X_), and the light-related parameters *n*, *m*, and *S*. The model parameters (*K*_X_, *n*, *m*, and *S*) were adjusted individually for each experimental condition to best reflect the respective cultivation parameters and light environment. These parameters were then used to generate simulated growth curves under nutrient-unlimited conditions, allowing for a direct comparison with the nutrient-limited experimental results.

In addition, the pH value of the culture broth was measured with a pH sonde WTW Multi 9620 IDS + SenTix 940, Xylem Analytics Germany Sales GmbH & Co. KG, Werilheim, Germany.

The biomass productivity Q_P_ was calculated using Equation (1), where P_begin_ is the biomass concentration at the beginning of the cultivation, P_final_ is the biomass concentration at the end of the cultivation, V_L_ is the culture volume, and t is the cultivation time.

(1) $$Q_{P}=\frac{(P_{final}-P_{begin})\cdot V_{L}}{t}\;(\mathrm{kg}\;h^{-1})$$

The analysis of the ammonium and phosphate content in the media was carried out according to DIN 38406 E5-1 and DIN 38405 D11-1, respectively [29]. A modified method of the total carbohydrate quantification developed by DUBOIS et al. [30] and Nielsen [31] was used to quantify the released EPS content at the end of the experiments within the media. The media was centrifuged to separate the media and biomass, and the remaining cell-free supernatant was utilised for the ammonium, phosphate and total carbohydrate quantification.

For quantifying the PC content within the biomass, a method developed by Bennett and Bogorad [32] and modified by the biotechnology laboratory of the Ostbayerische Technische Hochschule Amberg-Weiden was utilised. The purity was assessed with Equation (2) with A_615_ and A_280_ indicating the measured absorbances at wavelengths of 615 nm and 280 nm, respectively, and where higher values indicate higher purity.

(2) $$Purity=\frac{A_{615}}{A_{280}}$$

## 3. Results

### 3.1. Biomass Productivity

**Figure 2:**
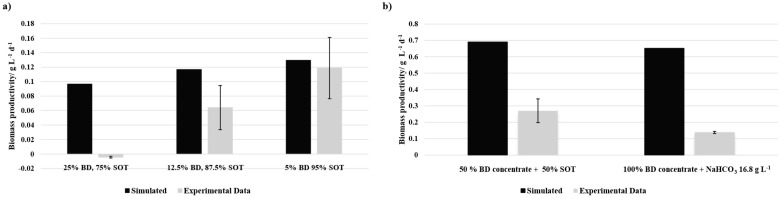
Experimental and simulated data of biomass productivity of (**a**) BD-SOT media and (**b**) media compositions with BD concentrate in batch mode. Error bars indicate the sample standard deviation of the (**a**) triplicate (*n* = 3) and (**b**) duplicate (*n* = 2).

**Figure 3:**
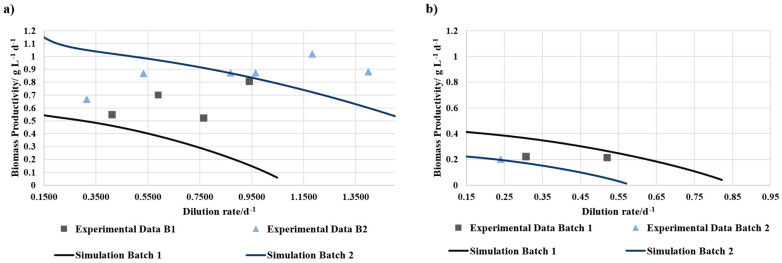
Biomass productivity of (**a**) 50% BD concentrate 50% SOT and (**b**) 100% BD concentrate + NaHCO_3_ in continuous operation mode over different dilution rates. The lines represent the simulated values for the individual trials, while the squares and triangles represent the experimental data.

The experiments were conducted under batch and continuous cultivation conditions. The flask batch experiment lasted approximately 300 h. The cultivation in the cylindrical bubble column reactor was carried out in batch mode (approximately 118 h) until the continuous process started. Regarding the batch process, the biomass productivity was assessed only at the end of the batch cultivation. In continuous mode, the biomass productivity was determined for each tested equilibrium point. Therefore, the biomass concentration of the equilibrium point was used for P and the dilution rate for the time. The results were compared with the biomass productivity determined by a growth model based on Jung et al. [28], which is based on light limitation. The data of the batch experiments are presented in Figure 2 and values of the continuous experiments are presented in Figure 3.

There was no growth on the media with 25% BD observable. Additionally, the biomass productivity in 12.5% BD was lower than in 5% BD. Compared with the simulated values, which are based on cultivation with SOT medium under the same conditions, adding BD to the media seemed to have an inhibiting effect on the growth. Hereby, the BD could be toxic in higher concentrations. The hypothesis of a nutrient deficiency can be dismissed in this instance. A lower rate of biomass productivity was observed in the 100% BD concentrate with NaHCO_3_ in comparison to both the simulation and the 50% BD concentrate. Also, 50% BD concentrate had a lower biomass productivity than the simulation during the batch mode. However, it seems like the nutrient balance in the media composition with 50% BD concentrate and 50% SOT media is better compared to the other tested media compositions. Additionally, the media compositions with the BD concentrate have no inhibition of the growth due to turbidity.

Figure 3 presents the biomass productivity of the experimental data compared to simulated data at different dilution rates. While 100% BD concentrate with added bicarbonate had lower biomass productivity and could therefore only be tested at low dilution rates, 50% BD concentrate showed at higher dilution rates similar or even higher biomass productivities than the simulated values. The differences within one trial may be due to different batches of BD concentrate.

### 3.2. Nutrient Uptake

#### 3.2.1. Phosphate Fixation

Phosphate concentrations were measured in the BD and BD concentrate prior to the cultivation and in all tested media compositions after the batch cultivation period. Here, phosphate fixation was observed only in the control group cultivated in standard SOT medium. In contrast, all other tested media compositions showed an unexpected increase in phosphate concentration during cultivation, suggesting a release rather than an uptake. As the biomass concentration at the end of the batch cultivation time was between 0 and 1.6 g L^−1^ and the phosphorus content of dry biomass is according to Brown and Shilton [33] approximately 1%, 0 to 0.049 g of phosphate should theoretically be fixated within the biomass. The absence of phosphate fixation could indicate a nutrient imbalance, which also explains the aforementioned lower biomass productivity compared to the simulated values. Additionally, the phosphate concentration in the media compositions with BD is 130–280% of the concentration of SOT media which can potentially be toxic for the cyanobacteria [26]. The accumulation was particularly noticeable in the medium 25% BD and 75% SOT and may be a result of phosphate released from the dead biomass.

**Figure 4:**
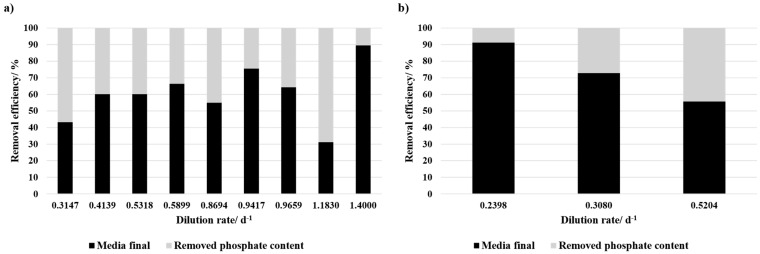
Phosphate removal efficiency at different dilution rates (d^−1^) during the continuous cultivation with (**a**) 50% BD concentrate and 50% SOT, and (**b**) 100% BD concentrate + NaHCO_3_. The black bars represent the phosphate concentration remaining in the medium after the experiment, while the grey bars indicate the removed phosphate content. A removal efficiency of 100% corresponds to complete phosphate depletion in the medium after cultivation.

In contrast, continuous cultivation facilitated phosphate fixation, with removal efficiencies for different dilution rates ranging from 8.8% to 68.8% (Figure 4). There was no clear correlation between the dilution rate and the phosphate removal detected for the cultivation with 50% BD concentrate. For 100% BD concentrate it seems like with increasing dilution rate the phosphate concentration within the media decreases. Consequently, the fixation efficiency increases. However, the value of the medium prior to the experiment is an average value of the different electrodialysis batches with differences in phosphate concentrations.

#### 3.2.2. Ammonium Fixation

To evaluate the ammonium removal efficiency in different media compositions, the ammonium content was measured before and after the batch cultivation as well as at each equilibrium point for different dilution rates during the continuous cultivation.

**Figure 5:**
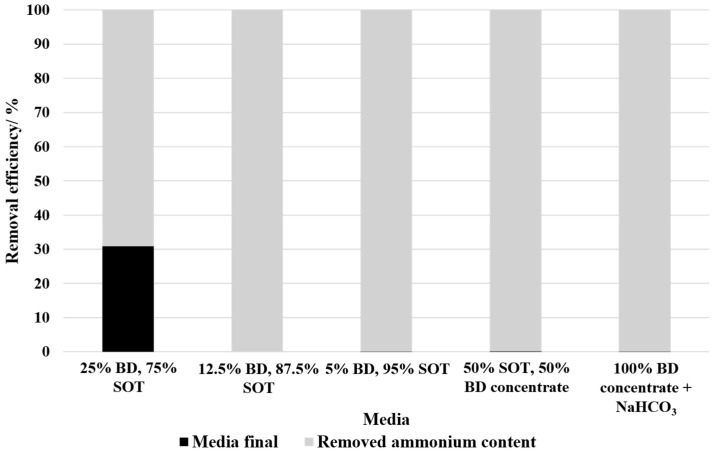
Ammonium removal efficiency of different media compositions. The black bars represent the remaining ammonium concentration in the medium after the experiment, while the grey bars indicate the removed ammonium content. A removal efficiency of 100% corresponds to complete ammonium depletion in the medium after cultivation.

Figure 5 presents the remaining ammonium concentration and removal efficiency for the tested media compositions in batch mode. Hereby, 25% BD showed the lowest removal efficiency with 69.15% despite no microbial growth. The rest of the media compositions showed a nearly complete ammonium removal with removal efficiencies of at least 99%.

During continuous cultivation, ammonium removal efficiency remained consistently high across all different dilution rates for both tested media compositions with BD concentrate. The removal efficiency of 100% BD concentrate was slightly lower with a minimum of 98% compared to the 50% BD concentrate with a minimum of 99% (Table 3).

### 3.3. Phycocyanin Yield

**Figure 6:**
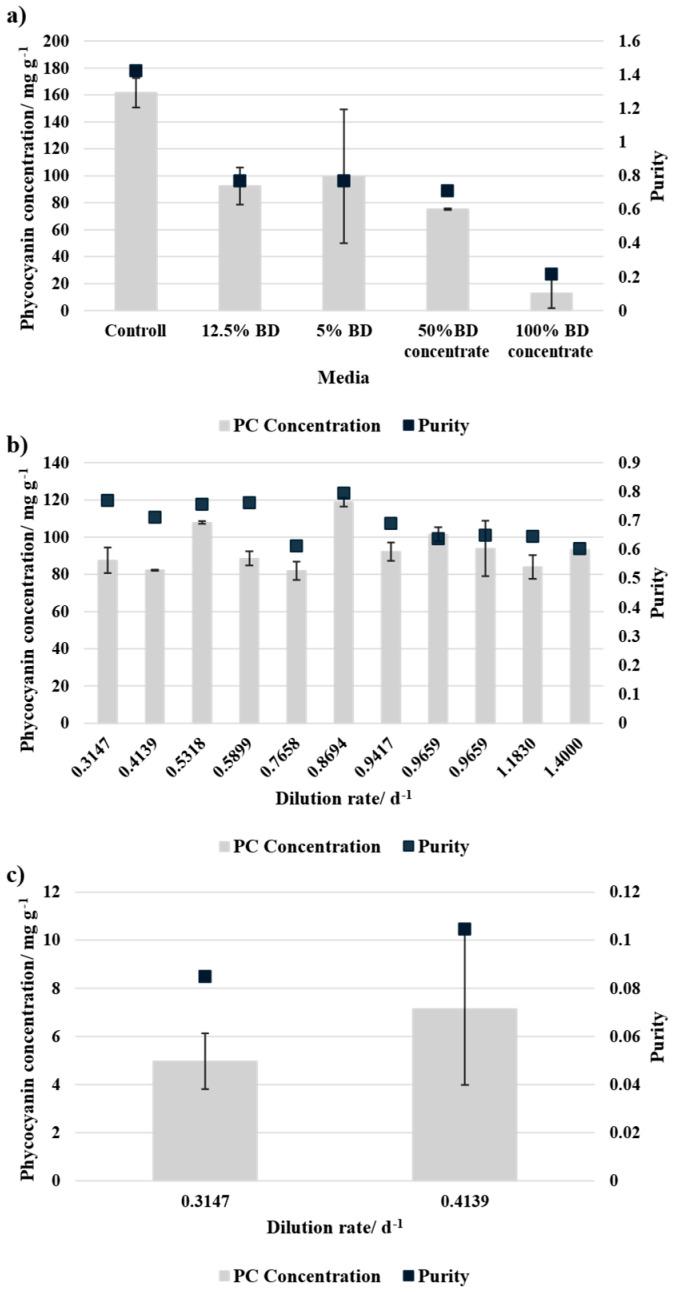
Phycocyanin concentration normalised to dry biomass and purity of the phycocyanin for different media compositions (**a**) after batch cultivation, and for each equilibrium point during continuous cultivation for (**b**) 50% BD concentration and 50% SOT, and (**c**) 100% BD concentration + NaHCO_3_. The error bars indicate the standard deviation of the measurement (*n* = 3).

The phycocyanin concentration was measured at the end of the batch cultivation and each equilibrium point of the continuous cultivation. The results were normalised to biomass dry weight to account for differences in culture growth. In batch cultivation, presented in Figure 6a, the control (SOT medium) exhibited the highest PC yield and purity. The cultures grown in 12.5% BD and 5% BD media showed similar values for PC concentration and purity. However, 50% BD concentrate resulted in a lower value for both concentration and purity compared to BD-based media. Notably, 100% BD concentrate showed the lowest phycocyanin content (<20 mg g^−1^) and purity, suggesting a lack of nutrients and therefore an inhibitory effect.

During the continuous cultivation, presented in Figure 6b,c, no clear correlation between dilution rate and phycocyanin concentration was observed. However, 50% BD concentrate results in higher PC concentration and purity compared to 100% BD concentrate and reaches slightly higher values compared to its batch cultivation. This indicates a better nutrient supply during the continuous cultivation mode.

### 3.4. Exopolysaccharides Yield

**Figure 7:**
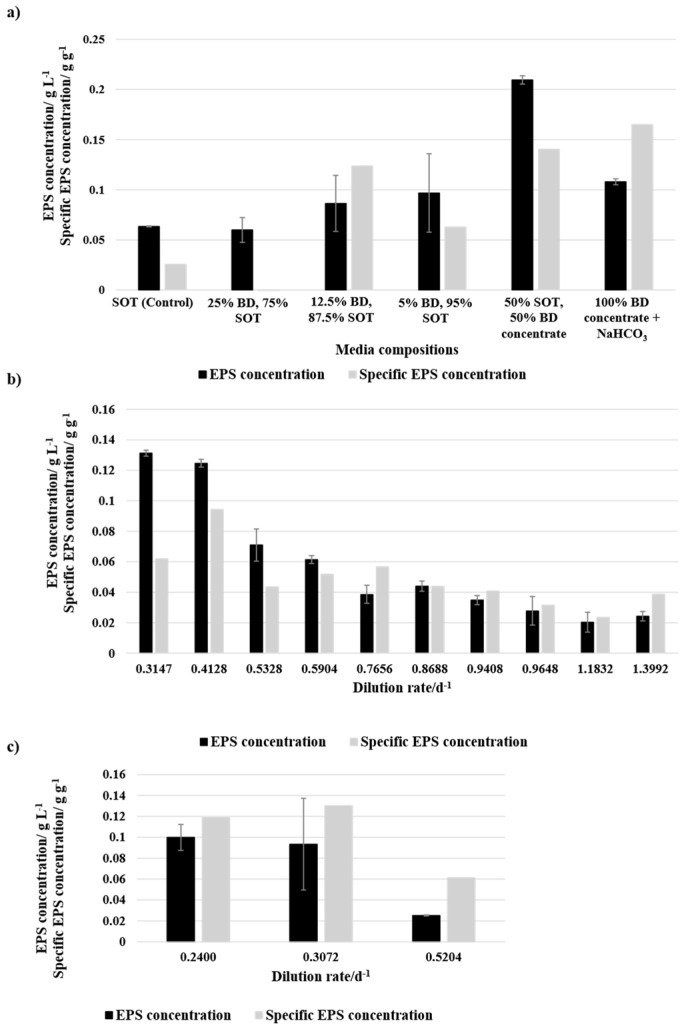
EPS concentration and specific EPS concentration of (**a**) batch mode and different media compositions, (**b**) continuous cultivation with 50% BD concentrate + 50% SOT and different dilution rates, and (**c**) continuous cultivation with 100% BD concentrate + NaHCO_3_ and different dilution rates. The error bars indicate the standard deviation of the measurement (*n* = 3).

The EPS concentration in the media was measured at the end of the batch experiments or an equilibrium point of the continuous process. Figure 7a presents the EPS concentration in the media and the specific EPS concentration per biomass after the batch cultivation of the different media compositions. The SOT (Control) and media compositions with BD were cultivated at approximately 33.4 °C and a light intensity of 75.7 μmol m^−2^ s^−1^. The media compositions with BD concentrate were cultivated at 35 °C and 180 μmol m^−2^ s^−1^. While 25% BD shows a similar EPS concentration of 0.0599 g L^−1^ to the control culture with 0.0634 g L^−1^, the specific EPS concentration was negative as there was no biomass productivity. The media compositions with 12.5% and 5% showed higher EPS concentrations with 0.0864 g L^−1^ and 0.0967 g L^−1^, respectively. Nevertheless, the specific EPS concentration of 12.5% BD was higher. As the culture with 25% BD was not growing, it cannot be guaranteed that the BD does not already contain sugars that are detected by the analysis. The EPS yield with the media compositions containing 100% BD concentrate showed similar EPS concentrations as 5% BD and 50% BD concentrate showed higher value even though the specific EPS concentration was lower compared to 100% BD concentrate. However, both media compositions with BD concentrate had, according to Jung et al. [34], optimal light and temperature cultivation conditions for EPS production.

Figure 7b presents the EPS concentration and specific EPS concentration of the equilibrium point of different dilution rates during the continuous cultivation utilising 50% BD concentrate + 50% SOT as media. Hereby, a trend of decreasing EPS concentration with an increasing dilution rate is visible. A similar trend can be observed for specific EPS concentration. Compared to an optimum 0.0138 g L^−1^ [34], similar values were reached with the lowest dilution rate of 0.0131 h^−1^, while 10% BD concentrate + NaHCO_3_ (Figure 7c) had lower concentrations compared to the value from the literature and 50% BD concentrate + 50% SOT.

### 3.5. Preliminary Cost Assessment of Different Media Compositions

To evaluate the economic feasibility of using BD and BD concentrate as alternative media components, a preliminary cost comparison was conducted against the artificial SOT medium. Table 4 presents an estimated cost breakdown for the tested media compositions in this study and an approximate profit assessment for the high-value products.

The sum cost reflects the expenses for each medium, including the cost of chemicals and, where applicable, the electricity costs associated with the electrodialysis process. The sum profit represents a theoretical revenue estimate based on the market value of the high-value products obtained from the cultivation process. This allows a direct comparison of input costs and potential financial return, providing an initial indication of economic viability.

The use of BD and BD concentrate has the potential to reduce media costs, as the chemical components of artificial SOT medium are expensive. Simultaneously, it is utilised as a wastewater treatment step. Nevertheless, this comparison is a basic economic estimate that does not account for operational expenses or processing costs. It is based on yield generated by experiments on a laboratory scale.

The costs for the media compositions with BD concentration are lower compared to the other media. Also, the yield for the produced high-value products is approximately the same compared to the control with SOT media. Consequently, this indicates the highest potential for saving costs.

## 4. Discussion

This study investigated the feasibility of using BD and BD concentrate generated from electrodialysis as alternative media compositions for the cultivation of *A. platensis*. Thereby, the formation of PC and EPS as high-value products were examined.

### 4.1. Biomass Productivity

Currently, no studies have been found that investigate the utilisation of BD and especially BD concentrate of biogas plants run with dairy wastewater as a composition for cultivation medium for *A. platensis*. This highlights the novelty of this study and the need for further research. However, research has been conducted on similar waste-derived media, such as piggery wastewater [38] or brewery wastewater [39,40].

Studies using wastewater as a cultivation medium often employ dilution with water to mitigate inhibitory effects like turbidity, high ammonium concentrations, or high concentrations of other inhibiting chemicals [16,38]. Moreover, BD is deficient in a carbon source, which also inhibits microbial growth. Consequently, in this study, the BD and BD concentrate were diluted with SOT medium instead of water, as a lack of nutrients was anticipated. To maintain a balanced cultivation environment and reduce inhibitory effects, a maximum of 25% BD concentrate was tested in the media compositions.

In batch cultivation, all BD-based media resulted in lower biomass productivity than the values simulated based on the model based on Jung et al. [28]. Among the diluted BD compositions, 5% BD yielded the highest biomass productivity, while 12.5% BD showed reduced productivity, and 25% BD failed to support microbial growth. The improved performance of BD concentrate-based media may be attributed to higher light intensities. However, despite this advantage, biomass productivity remained below simulated expectations, suggesting a nutrient imbalance may have affected growth efficiency.

In continuous cultivation, 50% BD concentrate achieved biomass productivities similar to or exceeded the simulated values, contrasting with its lower performance in batch mode. This suggests that nutrient replenishment in continuous mode may have alleviated some of the limitations observed in batch cultivation. In contrast, 100% BD concentrate exhibited unstable growth, which prevented an increase in the dilution rates. This suggests a clear lack of nutrients.

The ratios of C/N and N/P play a critical role in determining nutrient availability for the microorganism [26]. Only 100% BD concentrate had a significantly higher C/N and N/P ratio from SOT, suggesting that carbon or nitrogen limitation may not be an inhibitory factor in this media composition (Table 5). However, N/P ratios varied with 25% BD, 12.5% BD, and 5% BD all falling below the optimal range of 5–30 [26]. This suggests a leak in nitrogen sources, which is inhibiting microbial growth and consequently the biomass productivity.

The concentration of other nutrients and potentially toxic substances within the BD and BD concentrate used in this study are unknown. However, this could also influence the microbial growth. While BD provides a higher nutrient load, it may also contain organic compounds or heavy metals that disrupt microbial metabolism. Future studies should analyse BD and BD concentrate composition more comprehensively to determine which factors contribute to its effects on growth.

### 4.2. Nutrient Uptake

Phosphate fixation was absent in all tested media compositions during batch cultivation but occurred in continuous mode, with efficiencies ranging from 8.8% to 68.8%. This finding indicates that continuous nutrient replenishment may have a significant impact on phosphate uptake, as evidenced by the positive correlation between the availability of nitrogen and phosphate uptake [41]. Furthermore, adaptation to the two BD concentrate-based media during continuous cultivation has the potential to also influence the observed results.

Ammonium removal efficiencies were consistently high (>99%) across most media compositions in batch mode, except for 25% BD, which showed 69.15% removal despite no microbial growth, suggesting passive ammonium loss rather than biological uptake. In continuous cultivation, ammonium removal remained consistently high across all dilution rates, with 50% BD concentrate achieving >99% efficiency and 100% BD concentrate slightly lower, at a minimum of 98%. Consequently, the availability of nitrogen may be a limiting factor. However, media compositions mixed with SOT containing nitrate as an additional nitrogen source.

While high removal efficiencies of ammonium achieved in this study are consistent with the literature, the values for phosphates differ [38,42,43]. According to Bossa et al. [26], the removal of phosphate is dependent on different parameters like N/P ratio, CO_2_ concentration, and the species of microalgae. According to the other literature, *A. platensis* can effectively remove phosphate, and phosphate removal was detected during continuous cultivation. Consequently, a limitation of phosphate fixation due to the species can be excluded. However, in the present study, phosphate fixation was observed only in the control medium and continuous cultivation mode, while in the batch cultivation mode no fixation occurred for the tested media compositions. This may be attributed to metabolic changes induced by environmental stress. Araujo et al. [44] tested the nutrient fixation by feeding different voluminal of cassava wastewater to the cultures in feed-batch mode. Hereby, no fixation was detected when 2.0 mL day^−1^ was added and similar to this study the concentration of phosphate in the media increased. This phenomenon may be explained by phosphate release from dead or stressed cells, particularly since the measurements were taken at the end of the batch experiment when cultures had already reached stationary phase. As no intermediate sampling was performed, transient uptake followed by release cannot be ruled out.

In addition, during the experiments in this study, the cultures were aerated with ambient CO_2_, indicating a limited CO_2_ availability. Additionally, the N/P ratio was suboptimal in some of the media compositions, influencing the phosphate uptake. Additional experiments should be performed to investigate the influence of CO_2_ concentration on phosphate fixation. Furthermore, Khalaji et al. [22] showed in their study that the concentration of wastewater and inoculum concentration of the microorganisms influence the nutrient removal efficiencies.

According to Li et al. [45], the nitrogen content within the biomass of *A. platensis* varies between 4.5% and 10%. A calculation of the minimum and maximum N content in the biomass indicates that, for 4.5%, most of the media compositions exhibit passive ammonium loss rather than a complete fixation. Consequently, it is necessary to ascertain whether the ammonium was fixed within the biomass or if it was stripped due to elevated pH values and high temperatures [46,47].

### 4.3. Phycocyanin Yield

Phycocyanin was selected as a target high-value product due to its strong market potential and wide application in food, cosmetics, and pharmaceuticals [48].

PC production was strongly influenced by media composition. In batch cultivation, the control medium (SOT) achieved the highest PC concentration and purity. Cultures grown in 12.5% and 5% BD exhibited similar PC yields, whereas 50% BD concentrate resulted in lower PC concentrations and purity compared to BD-based media. The lowest PC yield (<20 mg g^−1^) and purity were observed in 100% BD concentrate, likely due to nutrient imbalances. In continuous cultivation, no correlation between dilution rate and PC yield was found, but 50% BD concentrate consistently outperformed 100% BD concentrate in both PC concentration and purity. In comparison to the batch cultivation, the PC concentration with 50% BD concentrate was similar or higher during continuous cultivation and for 10% BD concentrate even lower.

The reached values from the media compositions with 12.5% BD, 5% BD, and 50% BD concentrate are similar to Barati et al. [39] who used brewery wastewater for the cultivation of *A. platensis*. Slightly lower values were reported by Baraldi et al. [49] who utilised scotta whey, buttermilk, and dairy wastewater as substrates for the cultivation. Additionally, studies showed the direct impact of nitrogen limitation on the pigment content [45,50,51].

### 4.4. Exopolysaccharides Yield

EPS production was investigated as it plays a crucial role in various applications, including biomedicine, food and beverage production, and agriculture [52,53]. The selection of EPS as a target compound was based on previous findings confirming that the chosen *A. platensis* strain demonstrates consistent EPS production, allowing for a reliable assessment of its yield under biogas digestate-based cultivation [34,52].

EPS concentrations were highly variable across different media compositions. In batch cultivation, the EPS concentration in 25% BD was similar to that of the control (SOT). Due to the lack of microbial growth, the detected EPS may have originated from the BD itself or been actively secreted by cells in response to environmental conditions as EPS production is not necessarily correlated with biomass growth. Since EPS can exist in both slime and capsular forms, with the latter potentially being released under stress or changing environmental factors, distinguishing between externally derived and biologically produced EPS remains challenging [54]. Media compositions with 12.5% and 5% BD resulted in higher EPS concentrations, with 12.5% BD achieving the highest specific EPS concentration. 100% BD concentrate showed similar EPS concentrations to 5% BD, while 50% BD concentrate had the highest overall EPS concentration.

In continuous cultivation, 50% BD concentrate showed a decreasing EPS concentration with an increased dilution rate, following a similar trend for specific EPS concentration. The highest EPS yields were comparable to the literature values [34], particularly at the lowest tested dilution rate. However, 100% BD concentrate resulted in lower EPS concentrations than both the literature values and 50% BD concentrate.

Li et al. [55] describes the influence of nutrient availability on EPS production. Hereby, high concentrations of nitrogen sources and phosphate are likely to cause a stress response. Also, the type of nitrogen source influences the EPS yield [55].

### 4.5. Preliminary Cost Assessment

The cost analysis indicated that BD concentrate-based media compositions had significantly lower costs than the artificial SOT medium. Additionally, high-value product yields in these media were comparable to those in the control, suggesting potential economic benefits. Similar findings have been reported by Ma et al. [19], where microalgae-based wastewater treatment demonstrated cost-saving potential by eliminating the need for expensive synthetic media.

Furthermore, microalgae cultivation utilising wastewater streams such as BD and BD concentrate may contribute to lower energy demands and environmental impact compared to conventional treatment processes [56]. However, a more detailed economic assessment considering media production, process optimisation, and capital investment is required to determine the overall feasibility of BD and BD concentrate as sustainable alternatives.

### 4.6. Limitations

While this study provides valuable insights into the feasibility of using BD and BD concentrate as alternative media compositions for *A. platensis* cultivation, several limitations must be considered:

The chemical composition of BD and BD concentrate was not fully characterised, meaning the presence of potential inhibitory substances (e.g., heavy metals, organic acids, or high salt concentrations) remains unknown. A complete elemental and organic profile of BD could provide further clarity on its suitability as a cultivation medium and identify potential pre-treatment strategies to enhance its performance.

The electrodialysis process used to concentrate BD nutrients exhibited instability, leading to variations in nutrient composition between different batches. This inconsistency may have influenced the growth performance and product yields observed in BD concentrate-based media. Additionally, the overall nutrient transfer efficiency was low, with less than 23% of phosphate and ammonium from the biogas digestate being transferred to the BD concentrate. Future studies should focus on optimizing electrodialysis parameters to improve nutrient recovery efficiency and ensure a consistent nutrient profile in BD concentrate.

No pre-adaptation of *A. platensis* to BD-based media was conducted, which may have negatively affected growth performance at higher BD concentrations. Adaptation strategies, such as gradual acclimatization to increasing BD concentrations, could potentially enhance tolerance to inhibitory compounds and improve biomass productivity, phycocyanin yield, and nutrient uptake efficiency. Future studies should explore adaptive evolution approaches to assess the long-term potential of BD as a sustainable cultivation medium.

## 5. Conclusions

This study confirms the feasibility of using biogas digestate (BD) and electrodialysis-treated BD concentrate as alternative media for *A. platensis* cultivation. While 25% BD inhibited growth, diluted BD-based media (5–12.5%) supported biomass production and high-value product formation. Continuous cultivation with 50% BD concentrate achieved biomass productivities comparable to or exceeding simulated values, whereas 100% BD concentrate + NaHCO_3_ resulted in unstable growth due to nitrogen limitations.

Ammonium removal was highly efficient (≥98%), but phosphate fixation was primarily observed in continuous cultivation, likely due to N/P imbalances and lack of N. Phycocyanin yields in 12.5% BD, 5% BD, and 50% BD concentrate were comparable to the literature values, while EPS production peaked at 50% BD concentrate under low dilution rates. The preliminary cost assessment suggests BD concentrate-based media could be a cost-effective alternative to synthetic media. However, further research is needed to optimize nutrient composition, assess long-term stability, and evaluate large-scale feasibility.

## Figures and Tables

**Table 1:** Composition of Biogas Digestate and Electrodialysis Concentrate.

Parameter	Biogas Digestate (mg L^−1^)	Electrodialysis Concentrate (mg L^−1^)
Phosphate (PO_4_^3−^)	2251	30.4
Ammonium (NH_4_^+^)	611	145.3
Nitrate (NO_3_^−^)	1.5	-

**Table 2:** Composition of Zarrouk’s Medium (SOT).

Component	Concentration (gL^−1^)	Stock Solution
NaHCO_3_	16.8	Sol 1
K_2_HPO_4_	0.5	Sol 1
NaNO_3_	2.5	Sol 2
K_2_SO_4_	1.0	Sol 2
NaCl	1.0	Sol 2
MgSO_4_·7H_2_O	0.2	Sol 2
CaCl_2_·2H_2_O	0.04	Sol 2
FeSO_4_·7H_2_O	0.01	Sol 2
Na_2_EDTA·2H_2_O	0.08	Sol 2
A5-Solution	1.0 mL·L^−1^	Sol 2
H_3_BO_3_	2.86	A5-Solution
MnSO_4_·7H_2_O	2.5	A5-Solution
ZnSO_4_·7H_2_O	0.222	A5-Solution
CoSO_4_·5H_2_O	0.079	A5-Solution
Na_2_MoO_2_·2H_2_O	0.02	A5-Solution

**Table 3:** Ammonium concentration of 50% and 100% BD concentrate at equilibrium points of different dilution rates during continuous cultivation and their removal efficiency.

Dilution Rate (h^−1^)	Concentration of BD Concentrate	Initial Ammonium Concentration (mg L^−1^)	Final Ammonium Concentration (mg L^−1^)	Removal Efficiency (%)
0.0131	50%	72.63 ± 39.78	0 ± 0.0114	100
0.0172	50%	72.63 ± 39.78	0.015 ± 0.131	99.98
0.0222	50%	72.63 ± 39.78	0 ± 0.001	100
0.0246	50%	72.6 ± 39.783	0 ± 0.0704	100
0.0319	50%	72.63 ± 39.78	0.066 ± 0.004	99.91
0.0362	50%	72.63 ± 39.78	0 ± 0.0024	100
0.0392	50%	72.63 ± 39.78	0 ± 0.0109	100
0.0402	50%	72.63 ± 39.78	0 ± 0.0101	100
0.0493	50%	72.63 ± 39.78	0 ± 0.0084	100
0.0583	50%	72.63 ± 39.78	0 ± 0.0112	100
0.0100	100%	145.26 ± 39.78	1.351 ± 0.083	99.07
0.0128	100%	145.26 ± 39.78	1.730 ± 0.1547	98.81
0.0217	100%	145.26 ± 39.78	1.570 ± 0.0189	98.92

**Table 4:** Estimated cost for different media compositions and estimated profit.

Media	50% BD Concentrate + 50% SOT	100% BD Concentrate + 16.8 g L^−1^ NaHCO_3_	25% BD + 75% SOT	12.5% BD + 87.5% SOT	5% BD + 95% SOT	SOT	Sources
Electricity cost for Electrodialysis (EUR L^−1^)	0.0125	0.0250	-	-	-	-	[35]
Cost for chemicals (EUR L^−1^)	0.359	0.075	0.539	0.629	0.683	0.719	
Profit for biomass (EUR L^−1^)	0.014	0.006	0	0.007	0.015	0.024	[36]
Profit for PC (EUR L^−1^)	0.015	0.003	0	0.018	0.020	0.032	[37]
Profit for EPS (EUR L^−1^)	0.040	0.047	0	0.035	0.018	0.007	[37]
Sum cost (EUR L^−1^)	0.372	0.100	0.539	0.629	0.683	0.719	
Sum profit (EUR L^−1^)	0.069	0.055	0	0.061	0.053	0.063	

**Table 5:** Ratios of C/N and N/P of the different tested media compositions.

Media	C/N	N/P
SOT	5.82	4.63
25% BD, 75% SOT	4.21	1.71
12.5% BD, 87.5% SOT	5.00	2.48
5% BD, 95% SOT	5.49	3.43
50% BD concentrate, 50% SOT	4.57	5.31
100% BD concentrate + 16.8 g L^−1^ NaHCO_3_	21.26	11.41

## Data Availability

The data supporting the findings of this study are available from the corresponding author upon reasonable request. Requests for access to the data should be directed to Prof. Dr. Lindenberger at C.Lindenberger@oth-aw.de.

## Abbreviations

The following abbreviations are used in this manuscript:

BD	Biogas Digestate
C	Carbon
EPS	Exopolysaccharides
N	Nitrogen
OD	Optical Density
P	Phosphorus
P_beginn_	Product concentration at the beginning
PC	Phycocyanin
P_final_	Product concentration at the end
Q_P_	Biomass Productivity
V	Volume
